# Supplementary material for: Comparing the Efficacy of Multidisciplinary Assessment and Treatment, or Acceptance and Commitment Therapy, with Treatment as Usual on Health Outcomes in Women on Long-Term Sick Leave—A Randomised Controlled Trial
Source: Int J Environ Res Public Health. 2021 Feb 11;18(4):1754. doi: 10.3390/ijerph18041754 (PMC7916944; doi:10.3390/ijerph18041754)
Supplement: Supplementary file 1 [file ijerph-18-01754-s001.pdf]

Supplementary materials.

Methods

Further description of the TEAM intervention:

The physician was responsible for collecting the medical history and present health status, including reasons for sick leave, previous assessment and rehabilitation efforts. In addition, the psychologist or the physician conducted a psychiatric diagnostic screening with The Mini International Neuropsychiatric Interview (M.I.N.I.) and Montgomery-Åsberg Depression Rating Scale (MADRS-S), followed up on ongoing and previous psychological treatments; collected data for a behavioural analysis and explored the participant's motivation and values connected to important life areas; and assessed the needs and benefits of psychotherapy. The occupational therapist collected data on present or previous work experiences and conducted an activity analysis, based on the Canadian Occupational Performance Measure. The social worker assessed the social and economic situation of the participant as well as the need for further information and support regarding the social insurance system or other social welfare systems.

Interventions provided by the physician included further specialised medical assessment, referral to other specialists if needed, assessment of prescribed medicines and contacts with other physicians e.g. the physician that managed the sick leave certificate. The psychologist offered psychotherapy based on ACT as described by the single therapist protocol. Those who were offered ACT were also offered sessions outside the clinic on the same conditions as in the unimodal ACT intervention. The occupational therapist offered further assessments and efforts to develop, recover or maintain meaningful activities, also outside the clinic. The social worker offered social and economic counselling.

Results

Table S1. Means for imputed data.

Pain	Baseline	Six months	12 months
Control	6.43	6.63	6.08
ACT	6.25	5.70	5.00
TEAM	6.07	6.01	5.50
HADS anxiety			
Control	11.50	12.14	11.70
ACT	10.26	9.52	8.75
TEAM	10.53	9.50	8.08
HADS depression			
Control	9.85	9.93	10.01
ACT	8.45	7.55	7.35
TEAM	9.57	8.34	7.35
SWLS			
Control	13.88	13.74	13.42
ACT	16.76	17.05	17.05
TEAM	15.11	17.15	17.44
GHQ			
Control	15.52	15.70	15.41
ACT	18.43	19.82	19.87
TEAM	17.21	19.33	21.21

Note: ACT=Acceptance and Commitment Therapy; TEAM= Multidisciplinary assessment and multimodal treatment programme; HADS=Hospital Anxiety and Depression Scale; SWLS=Satisfaction With Life Scale; GHQ=Generalized Health Questionnaire.

Treatment specification ACT

Mean treatment duration in the ACT group was 156 days (SD = 79; range: 12-385). Mean number of treatment sessions was 9.6 (SD = 5.3; range: 1-23; Median = 9.0).

Treatment specification TEAM

For TEAM participants, an overview of (1) how the team assessed the need for further treatment from the specialists, as assessed by the multidisciplinary team and (2) the number of specialists that were involved in treatment, is presented in Table A2.

Table S2. Outcome of the TEAM Assessment of the Need for Further Involvement of the Specialists and Number of Specialists Involved in the Treatment Phase.

	Assessment outcome (n=85)	Involved in treatment (n=83)
Physician	33	37
Psychologist	66	56
Occupational therapist	62	53
Social worker	21	28
Number of specialists		
0	1	
1	19	22
2	36	34
3	19	24
4	10	3

When assessment was completed, a total of 83 participants (81%) started treatment with one or more specialists. Of the 83 participants that initiated treatment in the TEAM intervention group, 47 (57%) completed treatment according to the plan that was agreed upon following assessment, 23 (28%) participants completed treatment with at least one specialist and 13 (16%) participants did not complete any treatment. In the TEAM intervention, it was possible to change the course of the treatment during the time, based on the assessment of the therapist and request from the participants (see Figure 1). Hence, the treatment in the TEAM intervention was more flexible, in terms of possibilities for the team-members to reconsider the treatment plan and change or add modalities when deemed necessary or by request from the participant.

Treatment duration in the TEAM group was a mean of 240 days (*SD* = 133; range: 27-648). The mean number of sessions with the physician was 2.1 (*SD* = 1.6; range: 1-8; Median = 1), with the psychologist 8.9 (*SD* = 5.6; range 1-31; Median = 9), with the occupational therapist 3.4 (*SD* = 2.5; range: 1-9; Median = 2), and for the social worker 6.4 (*SD* = 6.2; range: 1-20; Median = 4). In the TEAM group, 12 participants received interventions for more than one year. In almost all of these cases, there was a need for further help from the social worker.
